# Analysis of Single-Step Pretreatments for Lignocellulosic Platform Isolation as the Basis of Biorefinery Design

**DOI:** 10.3390/molecules28031278

**Published:** 2023-01-28

**Authors:** Jhonny Alejandro Poveda-Giraldo, Maria Camila Garcia-Vallejo, Carlos Ariel Cardona Alzate

**Affiliations:** Institute of Biotechnology and Agribusiness, Department of Chemical Engineering, Universidad Nacional de Colombia sede Manizales, Manizales 170001, Colombia

**Keywords:** biorefineries, pretreatment efficacy, lignocellulosic fractionation, heuristic analysis, techno-economic analysis, social impact

## Abstract

Biorefinery feasibility is highly influenced by the early design of the best feedstock transformation pathway to obtain value-added products. Pretreatment has been identified as the critical stage in biorefinery design since proper pretreatment influences subsequent reaction, separation, and purification processes. However, many pretreatment analyses have focused on preserving and valorizing six-carbon sugars for future use in bioconversion processes, leaving aside fractions such as hemicellulose and lignin. To date, there has been no pretreatment systematization for the removal of lignocellulosic fractions. This work defines pretreatment efficacy through operational, economic, environmental, and social indicators. Thus, using the data reported in the literature, as well as the results of the simulation schemes, a multi-criteria weighting of the best-performing schemes for the isolation or removal of cellulose, hemicellulose, and lignin was carried out. As a main result, it was concluded that dilute acid is the most effective for cellulose isolation and hemicellulose removal for producing platform products based on six- and five-carbon sugars, respectively. Additionally, the kraft process is the best methodology for lignin removal and its future use in biorefineries. The results of this work help to elucidate a methodological systematization of the pretreatment efficacy in the design of biorefineries as an early feasibility stage considering sustainability aspects.

## 1. Introduction

The integral use of biomass for its subsequent conversion into high-value-added products is an approach associated with the biorefinery concept, where the product portfolio may comprise bulk and/or specialized chemical products [1]. The biorefinery concept starts from the analogy with crude oil refineries, where multiple products are generated from petroleum. To this end, several methodologies have been proposed for the conceptual design, optimization, and implementation of biorefineries [2]. Initially, a biorefinery should be understood as a network of facilities that integrates technologies, feedstocks, and equipment to transform biomass into products and energy [3]. However, other authors have proposed a broader definition specifying that *“a biorefinery is a complex system in which biomass is integrally processed or fractionated to obtain more than one product, including bioenergy, biofuels, chemicals, and high value-added compounds that can only be extracted from bio-based sources. The latter after a comprehensive study of the raw materials to be used and a sustainability analysis based on the latest state of the art technologies and approaches which include aspects of the three pillars of sustainability”* [1]. Therefore, the biorefinery design should include the evaluation of feedstocks, technologies and equipment, transformation routes, and products through technical, economic, environmental, and social analyses. The analysis of the dimensions is justified under the objectives of the biorefinery: (i) to maximize the value of the products; (ii) to reduce greenhouse gas emissions; (iii) to reduce dependence on non-renewable fuels; (iv) to stimulate rural development; and (iv) to promote the social welfare of the population [4]. The maximum use of biomass and the minimum production of waste, contributing economically, environmentally, and socially, is a sustainability challenge faced by the design of biorefineries. In this way, the objectives of biorefineries are framed in fulfilling the Sustainable Development Goals (SDGs) [5]. Additionally, different authors have established the direct relationship that the implementation of biorefineries has with the fulfillment of the 2030 agenda, framing biorefineries as a possible solution to multiple problems faced by society [6]. 

Different steps are involved during the biorefinery design by the early-stage approach: hierarchy, sequencing, and integration [7]. The hierarchization involves the global elements of the biorefinery, understood as feedstock flows, products, and transformation routes. The sequencing illustrates the logical order in which the unitary processes should be developed to maximize resource use. Finally, integration defines the possibilities of integrating raw materials, technologies, and products to obtain greater benefits [8]. Due to the diversity of biomass, social contexts, route and technology transformations, government policies, and local demand, biorefineries have become complex systems to analyze [9]. For this reason, a fundamental axis of biorefineries should be the contextualization of where the process will be developed. Therefore, preliminary localization studies are mandatory to benefit adjacent markets, considering the restrictions and identifying the possible economic areas that can be satisfied [10]. 

Biorefinery design involves different stages, starting with evaluating and selecting potential feedstocks. Then, the biomass is pretreated to recover or isolate its fractions to be further subjected to a combination of biological and/or chemical processes [1]. Based on the above and considering that biorefineries are established as a tool to promote the sustainability of production processes, biomass acquires relevance. Lignocellulosic biomass emerges as a fundamental axis to promote a sustainable society under the waste valorization transformed into value-added products [11]. Although biomass can mitigate certain pollution issues caused by fossil fuels, some authors have identified challenges related to availability, supply logistics, and conversion yields toward platform products [12]. The difficulties associated with the conversion processes show the importance of biomass conditioning, representing one of the most costly and relevant stages of the process [13]. Pretreatments involve physical and chemical changes in the biomass structure [11]. Physical pretreatments focus on increasing the surface area of the material to improve hydrolysis processes. Often, these structural changes are achieved by reducing particle size, increasing porosity, or altering structural regularity. For example, some authors suggest that using pretreatments such as ultrasound produces perforations in the biomass, increasing porosity and yield in enzymatic hydrolysis [14]. On the other hand, chemical pretreatments aim to generate ultrastructural and chemical modifications in the cell wall of the biomass. These modifications involve the fractionation of the polymers, which results in better accessibility by enzymes and better utilization of the lignocellulosic fractions [11]. The main components of biomass are cellulose, hemicellulose, and lignin coupled in a constitutive complex structure [15]. Currently, most applied pretreatments focus on recovering the cellulosic fraction for further conversion into biofuels [16]. However, in the design of biorefineries, pretreatments must seek the greatest benefit from all available lignocellulosic fractions to obtain value-added products from each one or their possible combination. For example, after diluted acid pretreatments, the xylose-rich fraction could be dehydrated for furfural production and used for agrochemical and solvent applications. Some studies have also studied the xylose valorization for xylitol production from steam-exploded corn straw due to the nutritional properties of this sugar alcohol [17]. Similarly, kraft pretreatment allows obtaining a black liquor with soluble lignin that can be fractionated for vanillin production. Pretreatments such as steam explosions make it possible to obtain concentrated cellulose with multiple valorization possibilities, such as the production of biofuels, bioproducts, and biosurfactants.

The main purpose of biomass pretreatment should be the access of biocatalysts to plant polysaccharides to be converted into platform products [18]. Additionally, some authors have reported that an adequate pretreatment should (i) decrease the enzymatic load necessary for the hydrolysis processes, (ii) avoid the loss of valuable fractions to platform products, (iii) minimize the generation of inhibitory compounds for the hydrolysis or fermentation processes, and (iv) allow the recovery of other fractions (such as lignin and hemicellulose) that can be converted into valuable co-products [18]. Thus, pretreatments become relevant in promoting the availability of lignocellulosic fractions and increasing the productivity of the process. For example, it has been reported that adequate pretreatment can increase the rate of enzymatic hydrolysis of lignocellulosic materials from 3 to 10 times [19]. Although different pretreatment performance studies have been performed, mostly focusing on cellulose isolation for biofuel production, no design factors have been involved in the scale-up stage focused on biorefineries. There are no in-depth technical, economic, and environmental assessments considering operational and scaling factors. In addition, there currently needs to be a clear systematization of pretreatments with applications in biorefinery design since effective pretreatment improves the performance of reaction and downstream processes. Therefore, the pretreatment processes in biorefineries should consider the integral valorization of lignocellulosic fractions, maximizing the isolation of fractions in both branched chains or platform products for further processing. This work aims to analyze the sustainability of pretreatment efficacy and its influence on the design of production processes in biorefinery schemes. Therefore, pretreatments for lignocellulosic isolation were assessed considering technical, economic, environmental, and social perspectives based on ten sustainable indicators. 

## 2. Results and Discussion

### 2.1. Pretreatment Screening

Table 1, Table 2 and Table 3 show the possible pretreatments that would remove or isolate each lignocellulosic fraction for future use in biorefinery schemes. It should be noted that the hemicellulose and lignin fractions are removed together in the liquor in most pretreatments. Hemicellulose is usually hydrolyzed to form five-carbon oligomers and monosaccharides such as xylose and arabinose, and a small six-carbon fraction such as galactose, glucose, mannose, and 4-O-methyl-d-glucuronic acid [20]. Therefore, this gives rise to the synthesis of by-products with industrial attraction due to their future processing for producing ethanol, lactic acid, and sugar alcohols such as xylitol, sorbitol, mannitol, ethylene glycol, and glycerol, among others. Unlike cellulose, hemicellulose has an amorphous and slightly random structure with low thermal and chemical stability, achieving more than 75% removal in aqueous hydrolysis at high temperatures [21]. This overview of hemicellulose hydrolysis and its by-products makes it a valuable candidate for the biofuel, food, and biomaterials industry. On the other hand, lignin is easily hydrolyzed by alkaline agents at high temperatures due to its amorphous and cross-linked structure together with hemicellulose [22]. Lignin pretreatments are focused on its isolation, and the degree of polymerization decreases, modifying the three-dimensional structure as it becomes less complex [23]. However, the aromatic structure of lignin, with functional groups including aliphatic, phenolic hydroxyl, and carbonyl groups, make it a potential raw material in the food, pharmaceutical, and perfume industries.

Initially, biorefineries were proposed to produce biofuels through the efficient use of biomass, focusing on cellulose valorization through enzymatic hydrolysis and further fermentation [24]. Therefore, pretreatments focused on cellulose preservation or isolation should constitute an essential part of the process design since some physicochemical, structural, and compositional factors of the biomass must be overcome. The molecular configuration and chain length cause cellulose to have a high degree of polymerization, which hinders enzymatic and microbial attack [25]. In addition, the cellulose structure has a large number of intramolecular and intermolecular hydrogen bonds due to the hydroxyl groups forming a superstructure (supramolecular structure) and hindering easy hydrolysis compared to hemicellulose and lignin [26]. That fraction of cellulose that can be removed after pretreatment belongs to the amorphous fraction of the polymer, resulting in soluble and insoluble cellulose molecules in the form of polymers and oligomers [27]. For example, it has been reported that after dilute acid pretreatment, the insoluble cellulose molecules have a degree of polymerization of 100-20,000, while the soluble one of 2-12 [28], or values of 500-1500 after kraft pulping [29]. Pretreatments have been described as alternatives to decrease the crystallinity while increasing the surface area, promoting the adsorption capacity of enzymes to the substrate [30].

The early selection of pretreatments was performed through the removal index ($R_{I}$). Although many criteria should be considered for pretreatment assessment, it is possible to identify some schemes that are not optimal for removing specific lignocellulosic fractions. It should also be noted that the operating conditions, summarized in the severity factor (Log ($R_{0}$)), drastically influence the $R_{I}$. For example, removals of 14.6–31.3%, 26.2–77.9%, and 29.7–93.8% can be achieved for cellulose, hemicellulose, and lignin, respectively, when the severity factor varies between 3.1 and 4.4 for SO_2_-catalyzed steam explosion pretreatment [31]. Even hemicellulose removals of 19.1–26.3% and lignin removals of 66.9–71.4% have been achieved when small severity factor variations of 0.2–0.7 were performed in recycled aqueous ammonia expansion (RAAE) pretreatments [32]. The information condensed in Table 1, Table 2 and Table 3 was based on a comprehensive review of the best single pretreatment schemes for lignocellulosic biomass. For cellulose isolation, it can be observed that wet air oxidation, diluted acid, and kraft pretreatments are the most effective in preserving the biopolymer, with values around 90–93% (or isolations of 7–10%). In contrast, ammonia fiber expansion (AFEX) and organosolv have been postulated for cellulose preservation as well as hemicellulose and lignin removal [33]; however, more than 19% of cellulose is hydrolyzed into the liquor as losses. Pretreatment screening should be based on the removal of the lignocellulosic fraction coupled with the accessibility degree ($A_{I}$) for further valorization. Although dilute acid and kraft pretreatment have cellulose removals of 10%, their $A_{I}$ is 57–62%, which can hinder bioprocesses that are inhibited by the presence of undesired compounds. For example, after dilute acid pretreatment, the water-insoluble solid (WIS) is constituted by cellulose and a large composition of lignin (cellulignin solids) that must be removed, as it inhibits bioconversions to ethanol or other bioproducts [34]. However, bioprocesses have been studied for the joint valorization of cellulose and hemicellulose-based substrates to avoid disposing of pentoses as waste, being the kraft process a candidate for these systems as it preserves 90% and 60% of the initial biopolymer respectively. For example, Mishra and Ghosh studied the fermentation of glucose and xylose after kans grass biomass fractionation with sulfuric acid for bioethanol production through a co-culture based on *Scheffersomyces shehatae* and *Zymomonas mobilis* [35]. Therefore, based on the $R_{I}$ and $A_{I}$ indexes, the kraft, organosolv, ionic liquid, dilute acid, RAAE, and wet air oxidation pretreatments were selected for cellulose isolation in the WIS.

Due to different operational (i.e., feed ratio, acid catalyst addition, temperature, residence time) and structural factors of the feedstock (i.e., recalcitrance, crystallinity, porosity, degree of polymerization), the pretreatments are not selective to a single fraction; instead, more than two fractions can be obtained in the hydrolyzed liquor. For example, acidification of biomass with SO_2_-catalyzed steam explosion and dilute acid has effectively decreased the crystallinity and degree of polymerization of lignocellulosic biomass while removing hemicellulose and small amounts of lignin [36]. Therefore, pretreatments can be repeated in lignocellulosic isolation schemes, such as kraft, which removes lignin while preserving a large amount of cellulose. For hemicellulose removal, steam explosion and diluted acid pretreatments are the best schemes for obtaining five-carbon platform products based on the $R_{I}$ and $A_{I}$ indexes. In steam explosion pretreatments, large amounts of xylose are generated, followed by aliphatic acids and furanic derivatives from the hemicellulose [37]. Aliphatic acids such as acetic acid are derived from the hydrolysis of acetyl groups, while other acids such as formic and levulinic acids are products of catalyzed thermochemical degradation. Acetic acid has been reported to decrease the pH of the liquor, stimulating the acid-catalyzed hydrolysis of the other components, and some of the pentose sugars are subsequently dehydrated to furfural and hydroxymethylfurfural (HMF) [34]. From Table 2, steam explosion, liquid hot water (LHW), organosolv, ionic liquids, dilute acid, and ammonia recycled percolation (ARP) schemes were selected as the best pretreatments for hemicellulose removal. On the other hand, lignin is usually insoluble in aqueous solutions and can be removed from the liquor, together with the hydrolyzed hemicellulose, through thermochemical treatments. Delignification reactions involve the cleavage of the non-phenolic β-O-4 bond and the α-O-4 phenolic bond [38]. Therefore, there is no selective pretreatment to lignin removal; instead, it is necessary to remove it from the liquor with hydrolyzed pentoses through other treatments. Normally, acidification steps are implemented by adding CO_2_ and inorganic acids, which promotes lignin precipitation. This precipitation can be explained by the colloidal nature of lignin, specifically as a hydrocolloid that precipitates at low pH due to the protonation effect of the acid groups of the lignin structure [39]. Based on the data reported in Table 3, the best lignin removal schemes were alkali, kraft, LHW, ionic liquids, RAAE, and organosolv pretreatments.

**Table 1:** Pretreatment screening for cellulose isolation.

Pretreatment	Raw Material	Operating Conditions			$R_{I}$ (%)			$A_{I}$ (%) ***	Reference
		Temperature (°C)	Pressure (bar)	Log (R_0_)	Cellulose	Hemicellulose	Lignin		
Kraft	*Eucalyptus globulus*	155	-	3.09	10.0	39.4	84.6	62.0	[40]
Organosolv	Wheat straw	160	-	3.37	19.6	93.4	62.5	77.9	[41]
Ionic liquids	Corn stover	160	-	4.02	15.5	81.5	69.2	75.4	[42]
Diluted acid	Bamboo green	180	-	3.83	9.9	98.8	16.6	57.7	[43]
RAAE *	Corn stalks	85	20.4	0.67	10.3	14.1	71.4	42.8	[32]
Wet air oxidation	Rice husk	195	5.0	3.79	7.1	75.5	97.3	86.4	[44]
AFEX **	Corn stover	130	44.8	2.06	27.8	34.6	23.5	29.1	[45]
Biological	Corn stalks	28	-	2.66	57.0	41.0	11.0	14.1	[46]
Biological	Switchgrass	28	-	2.29	22.0	14	24	81	[47]

* Recycled aqueous ammonia expansion; ** ammonia fiber expansion; *** calculated using Equation (3).

**Table 2:** Pretreatment screening for hemicellulose isolation.

Pretreatment	Raw Material	Operating Conditions			$R_{I}$ (%)			$A_{I}$ (%) ***	Reference
		Temperature (°C)	Pressure (bar)	Log (R_0_)	Cellulose	Hemicellulose	Lignin		
Steam explosion	Sugarcane bagasse	195	-	3.67	2.3	81.7	12.1	92.8	[37]
LHW *	Bermuda grass	170	-	3.84	29.8	88.8	33.8	68.2	[48]
Organosolv	Wheat straw	160	-	3.34	19.6	93.4	62.5	58.9	[41]
Ionic liquids	Switchgrass	160	-	4.02	15.5	81.5	69.2	57.6	[42]
Diluted acid	Bamboo green	180	-	3.83	9.9	98.8	16.6	86.8	[43]
ARP **	Corn stover	170	23.0	3.06	39.8	63.3	80.4	39.9	[49]
Biological	Hardwood	28	-	2.28	15.8	17.9	3.0	93.6	[47]
Biological	Wheat straw	28	-	2.48	16	94	49	67.5	[50]

* Liquid hot water ** ammonia recycled percolation; *** calculated using Equation (2).

**Table 3:** Pretreatment screening for lignin isolation.

Pretreatment	Raw Material	Operating Conditions			$R_{I}$ (%)			$A_{I}$ (%) ****	Reference
		Temperature (°C)	Pressure (bar)	Log (R_0_)	Cellulose	Hemicellulose	Lignin		
Alkali	*Eucalyptus camaldulensis*	150	-	3.25	6.7	33.3	63.6	79.9	[51]
Kraft	*Eucalyptus globulus*	165	-	3.69	17.2	51.0	97.4	65.9	[40]
LHW *	Wheat straw	190	-	3.95	41.8	92.3	64.9	32.9	[52]
Ionic liquids	Corn stover	140	-	3.43	49.4	56.9	94.4	46.8	[53]
RAAE **	Corn stalks	85	20.4	0.67	10.3	14.2	71.4	87.7	[32]
Organosolv	Wheat straw	160	-	3.37	19.6	93.4	62.5	43.5	[41]
ARP ***	Corn stover	170	23.0	3.06	39.8	63.3	80.4	79.9	[49]
Biological	Bamboo culms	60	-	2.28	8.8		53.3	95.6	[54]
Biological	Corn stover	28	-	2.66	58	51	64	45.5	[55]

* Liquid hot water; ** recycled aqueous ammonia expansion; *** ammonia recycled percolation; **** calculated using Equation (2).

### 2.2. By-Product and Inhibitors Formation

After pretreatment, by-products are obtained from the hydrolysis of macromolecules, especially hemicellulose and lignin, including five- and six-carbon sugars in the form of oligomers and monomers, aliphatic acids, furans, and phenolic compounds, among others. Each by-product can be used in other thermochemical and biochemical processes as raw material (platform product) to obtain value-added products or have an inhibitory effect in bioconversions. In hemicellulose hydrolysis pretreatments, mainly xylose and xylo-oligomers are produced from the cleavage of the glycosidic bond in the xylan chain, as well as small amounts of glucose, galactose, and arabinose [31]. Based on different bioconversion processes, xylooligosaccharides can be used as a platform product in the functional food, pharmaceutical, and chemical industries. Although xylooligosaccharides are not digestible by humans, they favor the proliferation of healthy microorganisms due to their prebiotic nature [56]. In general, oligosaccharides based on glucose, xylose, fructose, and galactose have been shown to be a potential partial substitute for sugars promoting the growth of bacterial flora and also for their antioxidant and antiallergic capacities [57]. Different pretreatments have been proposed for producing xylo-oligomers, highlighting LHW or autohydrolysis, acid, and alkaline hydrolysis [58]. On the other hand, xylose is an important industrial product as it can be used to produce xylitol, used in the food industry as a sugar substitute to produce diabetic-friendly or low-calorie products. Panjiar et al. have studied the production of biosurfactants from xylose-rich hydrolysates to reduce surface tension in oil-water mixtures in the food, pharmaceutical, and petrochemical industries [59]. The production of bioethanol [60] and xylonic acid, used as an additive in the food industry and precursor for producing green solvents and copolyamides [61], has also been explored.

The rich phenolic content of lignin makes it promising to use this heteropolymer in different industrial processes of food, pharmacy, and perfumery. Solubilized or precipitated lignin has been studied to produce vanillin as an additive in the chemical industry [62]. Other studies have focused on using lignin in producing new materials, such as phenolic resin, epoxy, and polyurethane, as emulsifying and carbon agents, among others [23]. The thermostability and mechanical properties of lignin mixed with thermoplastics such as polypropylene have been demonstrated [63]. Substituting phenol for lignin in phenol-formaldehyde synthesis has also been studied [64]. However, the main drawback is the high heterogeneity of the molecule and an efficient isolation method with high purity. Depending on the pretreatment employed, the structure of lignin may vary in terms of molecular weight, phenolic distribution, polydispersity, inter structural bonds, among others. As an alternative, fractionation through membrane filtration, solvents coupled with microwaves, or sequential precipitation has been studied [22]. Pretreatments with acid catalyst addition promote the formation of low molecular weight phenolic and aromatic compounds that can act as inhibitors in bioconversion processes, affecting both microbial growth and overall yield. This inhibition can be given to specific functional groups, as they can interfere with the cell membrane influencing its microbial function [34].

Furfural and HMF are also produced by the dehydration of monosaccharides at high temperatures. Furfural has gained importance as a solvent or as a precursor for the production of pesticides. In contrast, HMF has been widely used as an additive in petroleum-based polymer blends, such as polyester and polyurethane. Furfural and HMF inhibit yeast growth and decrease the yield and productivity of ethanol fermentations. However, anaerobic fermentations have been performed in recombinant transformants with xylose as substrate followed by the addition of furfural, leading to increased ethanol production due to the reduction of furfural to furfuryl alcohol to decrease the production of undesirable by-products such as xylitol [65]. Alkaline pretreatment has greater potential for fermentative processes as less inhibitory substances such as HMF and furfural are produced than acid pretreatment [66]. Other degradation by-products, such as aliphatic acids (i.e., levulinic acid, acetic acid, formic acid), can serve as end products or platform products for pharmaceuticals, plasticizers, and other additives. However, inhibition of these acids has been observed at concentrations higher than 0.1 M, as they can pass through the cell wall and dissociate, lowering the intracellular pH and leading to death [34,67].

### 2.3. Techno-Economic Analysis

The results of the technical analysis of the selected pretreatment schemes are summarized in Table 4. Regarding the technical aspect, the cellulose isolation pretreatment schemes showed no significant difference; only small yield changes for organosolv and ionic liquid were observed. Regarding hemicellulose, the yields were calculated based on the production of oligomers and five-carbon monosaccharides, with the diluted acid, organosolv, and LHW being the best schemes. It was observed that the ionic liquid was the worst scheme in terms of yield since the hydrolyzed liquor was submitted to ionic liquid recovery stages, preventing a good recovery of the hemicellulose carbohydrates. For the lignin removal yield, it was observed that the kraft process allows obtaining a greater amount of the heteropolymer, which was to be expected since it is the main methodology for obtaining soluble lignin in the black liquor. Likewise, in terms of energy, it can be seen in Table 4 that the schemes with the highest energy demand were the RAAE, ionic liquid, and organosolv pretreatments. This behavior is justified by using energy-demanding equipment such as distillation towers for solvent recovery in organosolv and pressurized equipment such as RAAE, among others. On the other hand, the pretreatment schemes with the lowest energy demand were LHW, dilute acid, and steam explosion. These pretreatments are characterized by short reaction times and low reagent demand. Thus, these pretreatments allow the isolation of the lignocellulosic fractions without high energy demands. However, some of these pretreatments require specific designs for their proper operation. For example, although steam explosion is not energy-demanding, it requires an operational design material resistant to high pressures or the impact between the high-pressure steam and the biomass, or the dilute acid requires a specific alloy construction that withstands the acidic nature of the medium and does not corrode the reactor and its accessories. Therefore, an economic analysis of the operating and investment costs of each pretreatment scheme is essential.

Table 5 summarizes the economic results regarding investment (CapEx) and operating (OpEx) costs. In terms of CapEx, the most expensive pretreatment schemes were organosolv and RAAE, which can be explained by their use of complex equipment such as the distillation system (i.e., the organosolv scheme), which includes the use of the column, condenser, reboiler, reflux pump, and collection tank. In contrast, the most economical processes were LHW, dilute acid, and kraft. These processes were characterized by using few operating units and mild operating conditions, resulting in the lowest OpEx. On the other hand, the pretreatments with the highest OpEx were the ionic liquid and RAAE processes. Expensive reagents characterize the ionic liquid process, and its recirculation is crucial. To reduce the OpEx in the ionic liquid schemes, recirculation of the ionic liquid was proposed by adding anti-solvent to the reactive mixture in a 1:1 by weight ratio followed by centrifugation and evaporation to remove the anti-solvent from the ionic liquid for its subsequent recirculation [68]. This process allowed more than 10% decrease in OpEx associated with the demand for reagents. Finally, the organosolv and RAAE pretreatments showed a high demand for OpEx due to the need for utilities during the process, specifically electricity and steam.

**Figure 1:**
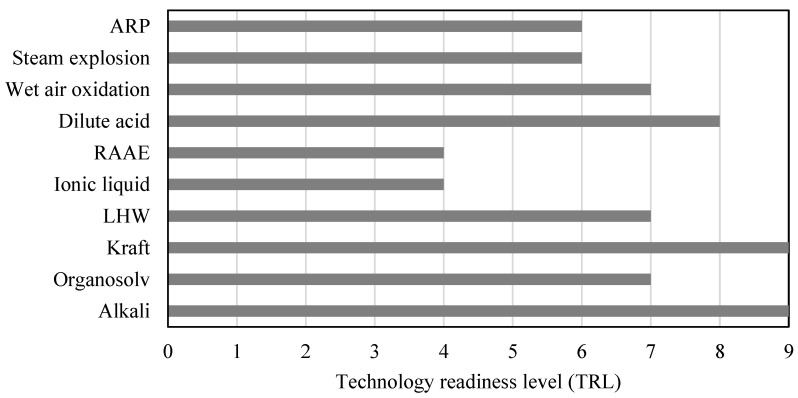
Technology readiness level of the selected lignocellulosic pretreatments.

The pretreatment selection should involve operational aspects and the overall performance of the process. It is also necessary to identify scaling factors or technology development level since, in many cases, its implementation on an industrial scale depends on technical, economic, and environmental design factors. Therefore, the most appropriate selection of the isolation scheme should involve the technology readiness level (TRL) since readiness defines whether the technology is applicable at the industrial or pilot scale, as well as whether it is in the innovation and development stage. Figure 1 shows the TRL results for the selected processing schemes for each lignocellulosic fraction. It can be observed that the high capital cost of pretreatments, such as RAAE, limits investors from using them on an industrial scale. Pretreatments such as organosolv involve solvents that increase the energy requirements and operating costs of the process, decreasing its economic viability. The economic feasibility of organosolv schemes has been widely reported for lignin extraction at the pilot scale [69]. The kraft and alkaline processes are used at the industrial scale for removing lignin from woody biomass for pulp and paper production. Although dilute acid pretreatment has been widely discussed in biotechnological applications, its implementation at the industrial scale has been slowed down. These delays are largely due to the generation and accumulation of inhibitory compounds and the high corrosion of processing units and piping [69]. Therefore, TRL must be analyzed from an operational and design perspective with techno-energetic and economic considerations.

### 2.4. Environmental Analysis

**Figure 2:**
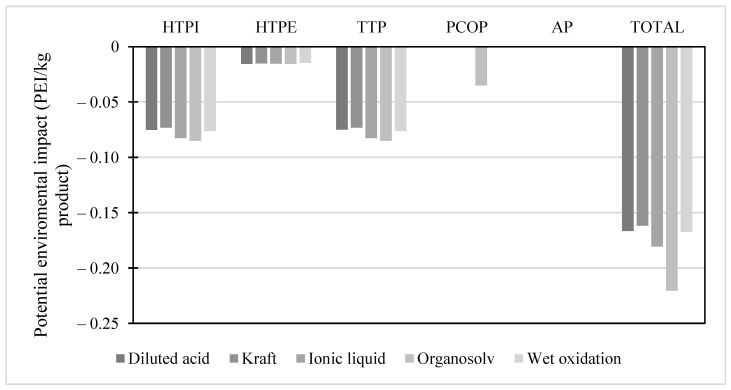
Potential environmental impact of pretreatment schemes for cellulose isolation.

The potential environmental impact of the pretreatment schemes during the isolation of the cellulose fraction is shown in Figure 2. It can be seen that all pretreatments presented similar impacts in the categories analyzed. Therefore, positive environmental impacts were observed, given that their values are negative, interpreted as environmental relief. Additionally, it can be seen that in the photochemical oxidation potential (PCOP) category, the organosolv pretreatment generated less impact because, during pretreatment, there were no considerable emissions of polluting gases, dust, or smoke. Although there could have been impacts related to human toxicity by ingestion (HTPI), there was no negative environmental impact since more than 95% of solvents are recirculated to the process. On the other hand, the RAAE pretreatment is not considered in Figure 2 since its potential presented considerable differences concerning the other pretreatments. The RAAE generated a harmful environmental impact, with 106 PEI kg^−1^ of product. This impact was reflected in higher rates in the HTPI and terrestrial toxicity potential (TTP) categories since they involve effects on humans either by ingestion, inhalation or dermal exposure to ammonia

**Figure 3:**
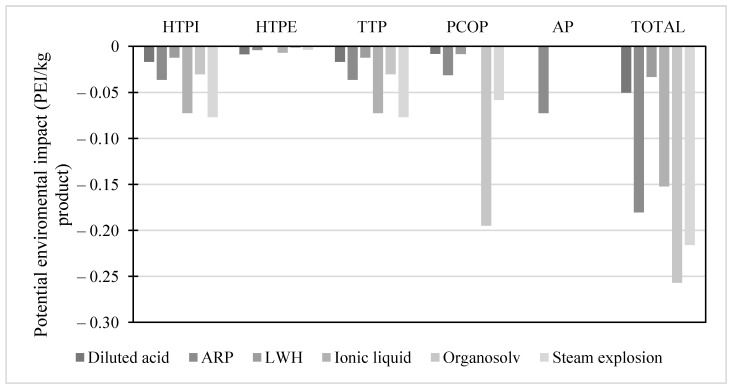
Potential environmental impact of pretreatment schemes for hemicellulose isolation.

The environmental impact of the pretreatment schemes for the isolation of the hemicellulose fraction is presented in Figure 3. The pretreatment schemes that exhibited the greatest environmental benefit were the ionic liquid and steam explosion in the human impact categories and the greenhouse gas emissions. Therefore, the organosolv pretreatment, although it contemplates using solvents, does not present pollutant gas emissions due to the recovery or recirculation of the solvent. This analysis also applies to the ionic liquid pretreatment, with the recirculation of the main process reagent. On the other hand, the ARP pretreatment showed the lowest favorable index due to the use of ammonia during the reactive process. Different authors have previously reported the effects of ammonia on the ecosystem and human health [70]. Thus, organosolv pretreatment and steam explosion are the best environmental schemes for hemicellulose recovery.

**Figure 4:**
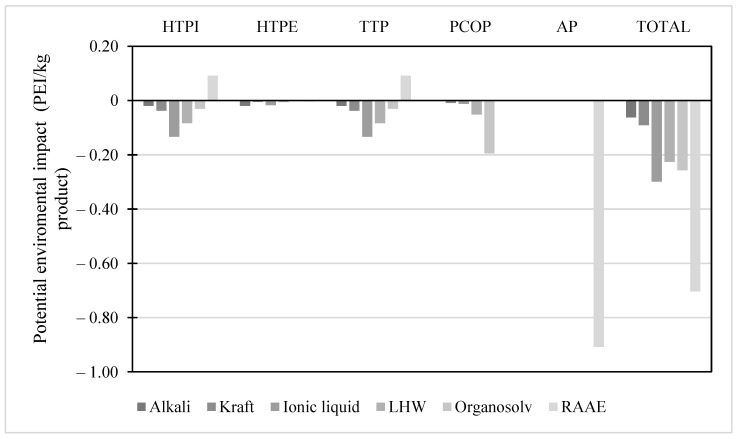
Potential environmental impact of pretreatment schemes for lignin isolation.

The potential environmental impact generated by the pretreatment schemes for the isolation of the lignin fraction is shown in Figure 4. Similar to the pretreatments for cellulose isolation and hemicellulose removal, the schemes used for lignin fractionation did not express any harmful environmental impact, except for the RAAE pretreatment concerning human toxicity. The RAAE pretreatment is the only pretreatment that shows harmful environmental impacts for pretreatments focused on cellulose and lignin recovery. This phenomenon is associated with the high ammonia concentration in the reactive mixture. These concentrations reach values of more than 50% *v*/*v*. Therefore, downstream ammonia removal becomes a complex process, considering subsequent separation stages. In addition, the lignin obtained from pretreatments such as RAAE cannot be used in food-type production schemes because the contaminant traces cannot be eliminated.. The impact associated with using hazardous substances during the process triggers complications in the development of pretreatment since it requires the installation of extensive and rigorous safety protocols.

### 2.5. Social Analysis

For the social analysis, a comparison of indicators contextualized by the capacity in Colombia was performed. These ratios considered statistics at the national scale for certain economic sectors. Based on water consumption in pretreatment schemes for cooling and processing, two indicators were calculated: (i) water for the industrial sector, where Colombia demanded 3.73 × 10^9^ m^3^ in 2019, and (ii) total water available in the country, with reported values of 2145 × 10^9^ m^3^ in 2019 (AQUASTAT). Likewise, indicators referring to the demand for electricity from the processing units were analyzed with the national electricity capacity of 72,824 GWh in 2021 and the consumption of diesel (in energy terms) for the generation of thermal energy from the different steam qualities of the pretreatment schemes with the amount of energy provided by Colombia in 2021 (2.40 × 10^11^ MJ) (UPME). For the latter analysis, a boiler thermal efficiency of 80% was assumed. Table 6 shows the results of the indicators analyzed for each lignocellulosic fraction. It can be seen that for all indicators, except for the level of industrial water use (withdrawal), the values are very small since they are contextualized to the country. Therefore, the comparison and analysis were carried out between the different schemes and not based on a risk scale. From the perspective of the level of industrial water use (withdrawal), it can be observed that the organosolv pretreatment, in the three lignocellulosic fractions targeted, consumes the most water in the industrial sector. The addition of the dilute solvent during hydrolysis, the cooler prior to filtration of the hydrolysate, and the condensation of the solvent in the recovery stage represents the greatest social risk for its implementation. It was expected that pretreatments such as steam explosion would not greatly affect industrial water use (renewable) since small feed ratios between steam and biomass are required. Regarding electricity energy demand, the RAAE, LHW, and kraft pretreatments are the most at risk for cellulose isolation and removal of hemicellulose and lignin. Finally, from the perspective of fossil fuel extraction, there was no significant impact on the national diesel data. This shows that there is no great dependence on this fuel in boilers for steam production, and it does not represent any risk due to competition in its use. The low consumption of process water in steam explosion means that low levels of steam and diesel are required, making it the scheme with the lowest risk.

### 2.6. Pretreatment Efficacy

Based on the results of each evaluative indicator, an overall assessment of pretreatment efficacy was carried out by weighing the heuristic analysis. Heuristic analyses have been described as subjective analyses based on theoretical, experimental, or simulated results. Some investigations have shown satisfactory results of raw material selection [71] or transformation routes [72] through manual weightings. Therefore, the efficacy calculation contemplated the weighting of two types of ratings (see Table 7): (i) weighting by equal assignment of 10% for each indicator ($\omega_{1}$); and (ii) weighting by statistical analysis ($\omega_{2}$). As can be seen, despite the use of statistical weighting, where there is greater relevance to economic design indicators appealing to investors, there are no differences between the efficacy results compared to equal distribution. On the other hand, Table 7 shows that dilute acid, wet air oxidation or organosolv pretreatment is recommended to isolate the cellulose fraction. It should be noted that after each pretreatment, it is necessary to rinse the WIS to remove the greatest amount of unwanted soluble compounds or the excess of initial chemical reagents. In biorefinery schemes, the cellulose should be as clean as possible since degradation compounds such as furans or phenolics inhibit the bioconversion processes. It is recommended to perform neutralization stages with lime to neutralize and detoxify the solid stream, whose reaction product, such as calcium sulfate or gypsum, has been used as a co-product in biorefineries based on lignocellulosic biomass [73]. On the other hand, to remove hemicellulose in five-carbon sugar platform products, LHW, steam explosion, or dilute acid should be used, while to remove lignin heteropolymer, kraft, alkali, or LHW pretreatment is recommended. Many authors have previously reported that pretreatments are efficient when there is the direct formation of sugars or after enzymatic processes with the least loss of sugars, the formation of inhibitory compounds is limited, and energy demand and operating costs are minimized [74]. Other research has focused on the fact that pretreatments should remove lignin and hemicellulose, as well as reduce cellulose crystallinity and increase biomass porosity [27]. Although these definitions have already been discussed, many focus on the maximum cellulose utilization for bioconversion processes aimed at biofuels, leaving aside the other lignocellulosic fractions. The present work focuses on a more exhaustive assessment involving more evaluative indicators of operability, energy demands, profitability, and environmental and social impact. Therefore, a first approximation of the sustainability of pretreatment in biorefinery designs is given through technical, economic, environmental, and social assessment pillars. For example, under controlled conditions, dilute acid is sustainable because it generates a large amount of hydrolyzed five-carbon sugars while preserving cellulose, its TRL is high for industrial application, it has low energy demand, low investment and operating costs, a positive environmental impact, and good indexes of access to material resources in the social sphere.

The single-step pretreatment efficacy results give a tentative route to the future reaction and downstream processes that should be involved in the early design of biorefineries. Although the heuristic analysis provides design support, there are certain challenges to be considered in the biorefinery approach. (i) Insufficient separation of hemicellulose and lignin in the hydrolyzed liquor requires additional steps for lignin precipitation through acids, whose concentration can alter the soluble carbohydrates to form degradation compounds. (ii) In pretreatments that are acid-catalyzed, such as dilute acid, organosolv, or steam explosion, extra design factors must be considered due to the high temperatures used together with the corrosive action of the acids. Therefore, hot acid corrosion-resistant alloys must be used for reactor design, which makes the cost of the reactor, exchangers, filters, and piping an important element of the CapEx as well as possible maintenance costs. (iii) The main demand for process water in biotechnological processes comes from the pretreatment stage due to the high feed ratios needed to hydrolyze the lignocellulosic matrix, which sometimes makes it necessary to add costly chemical reagents that are difficult to recover and that alter the environmental impact of the waste streams. However, processes such as organosolv and kraft have been implemented at high scales considering the recovery of reagents. For example, green liquor causticization of the kraft process after the black liquor incineration. (iv) High energy use in the first stage of the process (pretreatment), such as organosolv. Therefore, it is proposed to contemplate energy integration stages to reduce the demand and costs associated with thermal energy.

During biorefinery design, the integral use of raw materials to produce high-value-added compounds is essential. Moreover, the objective is to minimize the environmental impact and costs associated with waste generation and maximize profits through the production of more compounds. Therefore, the design of pretreatment schemes through efficacy should involve using the other lignocellulosic fractions that were not considered a target product. For example, alkali pretreatment for lignin removal was studied without contemplating the future utilization of cellulose-rich WIS, followed by unhydrolyzed hemicellulose. Therefore, the pretreatment efficacy study may involve additional schemes sequentially to utilize each fraction best. Since pretreatment is the critical stage in the design of biorefineries, as explained by the onion diagram, it is required to optimize the processes to maximize the production of platform products. Different authors have studied sequential pretreatments to improve operationally future processing steps, such as enzymatic hydrolysis [75], buffering problems in the LHW [76], and decrease inhibitory compounds and residence times [77].

## 3. Methodology 

The pretreatment assessment in biorefinery schemes was performed based on indicators involving literature data and simulation results. This assessment began with selecting a processing objective: to identify pretreatments that best isolate each lignocellulosic fraction separately for further valorization. A screening of pretreatment schemes was performed considering composition and fraction removal constraints. Afterward, ten indicators were described and assessed, involving technical, economic, environmental, and social aspects. Finally, each indicator was scored based on data from the literature and simulation depending on what is most suitable in the final biorefinery design to improve the sustainability of the process. The best pretreatment schemes were selected based on the efficacy results performed by the heuristic analysis.

### 3.1. Pretreatment Screening

A literature review was performed to determine the best pretreatments to isolate each lignocellulosic fraction individually, either as a water-insoluble solid (WIS) or in the pretreatment liquor, considering thermal, chemical, and thermochemical processes. The screening involved more than 100 updated review and research-type papers (articles from 2018 to 2022) concerning pretreatments with experimental and simulation sections. Biomass containing a lignocellulosic compositional range of 25% < cellulose < 50%, 20% < hemicellulose < 40%, and 10% < lignin < 35% was chosen to restrict the analysis scope to comparable raw materials by composition. After the screening, six pretreatments were selected that best removed or isolated the fractions of interest based on the removal index ($R_{I}$) (see Equation (1)), resulting in a total of 18 schemes: six for cellulose, six for hemicellulose, and six for lignin. Pretreatments with removals to liquor higher than 60% or conservations higher than 80% in the WIS were considered.

(1) $$Removal\left(R_{I}\right)=\left(1-\frac{Pretreated\;fraction_{dry\;basis}}{Raw\;feedstock_{dry\;basis}}\right)\times 100\%$$

### 3.2. Efficacy Assessment

The pretreatment efficacy in biorefineries was assessed based on ten evaluative indicators to obtain the isolated lignocellulosic fraction or a platform product from the fraction of interest. The indicators were classified as technical (indicators (i)–(vi)), economical (indicators (vii) and (viii)), environmental (indicators (ix)), and social (indicator (x)). (i) Lignocellulosic fraction removal ($R_{I}$). This factor involves the lignocellulosic fractional amount isolated in the liquor or in the WIS after pretreatment (see Equation (1)). For lignin and hemicellulose, high individual removals are desired in the hydrolysate, while cellulose should be minimized. (ii) Accessibility index $(A_{I}$). During pretreatment, isolating only one fraction without altering the others is impossible. Therefore, the $A_{I}$ represents the accessibility rate of the isolated fraction affected by the remaining fractions or the contamination by undesired fractions. For valorization in the hydrolysate (see Equation (2)), high $R_{I}$ of undesired fractions lead to liquor contamination (leading to low $A_{I}$), whereas the fraction valorization in the WIS (see Equation (3)) requires high $R_{I}$ of undesired fractions (leading to high $A_{I}$). (iii) Formation of by-products or hydrolyzed products (${HP}_{I}$). Many oligomers, monosaccharides, and carboxylic acids are produced from the hydrolysis of the representative biopolymer biomass [78], which can be considered platform products in biorefineries for further processing. Thus, higher by-product formation implies higher valorization proposals to improve biorefinery profitabilities. (iv) Formation of inhibitory products $({IP}_{I}$). This indicator involves the number of degradation products inhibitory to biochemical processes, such as furan compounds, and their removal is essential [79]. Thus, minimal ${IP}_{I}$ values are desired for pretreatments. (v) Technology readiness level (${TRL}_{I}$). Many of the pretreatments are applied at laboratory or pilot scale since their scaling up is complex due to design and operability factors, cost, and energy demand. Therefore, the ${TRL}_{I}$ demonstrates the progress or applicable extent of research and scaling up of the technology, as shown in Table 8. (vi) Energy demand $(E_{I}$). Utilities, such as steam, cooling water, and electricity, can be expressed as annual operating expenditures (OpEx). Therefore, those processes where energy demand is minimized are the most promising for scale-up. (vii) Capital cost (CapEx) and (viii) OpEx. Higher technological complexity of processing units and sub-units increases the investment cost, decreasing its attractiveness for investors. Therefore, these indicators relate to the total investment cost of the pretreatment system and the operating costs described as raw material, utilities, labor, maintenance, and depreciation, which must be minimal for economic feasibility. (ix) Potential environmental impact (PEI). Many pretreatments involve chemical additives that are harmful to both humans and the environment, as well as corrosive, and may damage the durability of the processing units. Therefore, the PEI considers the environmental impact of pretreatment that should be minimized. (x) Social impact (SI_I_). This indicator involves comparing energy and water consumption to national or regional availability, providing a perspective of the level of risk to the communities surrounding the biorefineries that involve the pretreatment schemes. The energy and water demand of the process must be minimized so as not to affect the energy grid and water sources in the country.

(2) $$Accessibility\;index\;in\;the\;liquor\left(A_{I}\right)=100\%-\left(0.5\;R_{I}\;of\;undesired\;fraction_{1}+\;0.5\;R_{I}\;of\;undesired\;fraction_{2}\right)$$

(3) $$Accessibility\;index\;in\;the\;WIS\left(A_{I}\right)=0.5\;R_{I}\;of\;undesired\;fraction_{1}+\;0.5\;R_{I}\;of\;undesired\;fraction_{2}$$

Pretreatment efficacy was assessed using a quantitative approach to heuristic analysis methodologies [71]. This score was calculated manually based on literature data and used to estimate the values of indicators (i)–(v) and as inputs for the simulation schemes, such as for indicators (vi)–(x). The assessment considered a rating from 1 to 10 for each efficacy indicator, where 1 represents the lowest score and 10 the highest. For the ${TRL}_{I}$ indicator, normalizations to a scale of ten were performed according to the data in Table 8. The ratings were weighted to obtain a comprehensive indicator of each pretreatment based on the relevance of each parameter (weight factor) using Equation (4), where $\omega_{i}$ is the weight factor and $I_{i}$ the evaluative indicator. The weight factor was calculated as the ratio of the specific variability ranges for each indicator over the best-case scenario, as suggested elsewhere for multi-criteria decisions in biorefineries [80].

(4) $$Efficacy=\sum_{i}^{n}\omega_{i}\times I_{i}$$

### 3.3. Simulation Procedure

For the assessment of the $E_{I}$, CapEx, OpEx, PEI, and SI_I_ indicators, the pretreatment schemes were simulated in Aspen Plus v9.0 software (Aspen Technologies, Inc., USA), considering production yields, removals, operating conditions, and feed ratios described in the literature. A processing flow rate of 50 tons d^−1^ of rice husk and a simulation scope up to filtration or separation of liquor and WIS fractions were assumed. The raw material characterization is presented in Table 9. The chemical and thermodynamic properties of cellulose, hemicellulose and lignin were specified based on that reported by the National Research Energy Laboratory (NREL) [81]. The properties and chemical equilibria of the liquid and vapor phases were estimated through the Non-Random Two Liquids (NRTL) thermodynamic method and the Soave–Redlich–Kwong equation of state, respectively.

#### 3.3.1. Techno-Economic Assessment

The overall pretreatment yields were calculated for each lignocellulosic fraction based on the initial raw material flow rate. For the cellulose yield, the six-carbon total content in the WIS was considered, as shown in Equation (5). The oligomers and monosaccharides of five carbons were used for hemicellulose yield (see Equation (6)). Meanwhile, Equation (7) involves the solubilized lignin in the liquor fraction. On the other hand, utility requirements were calculated for steam, cooling water, and electricity. For the CapEx analysis, the direct cost of the processing equipment was estimated using the Aspen Process Economic Analyzer v9.0 software (Aspen Technologies, Inc., USA) and based on the mass and energy balances of the simulations. The CapEx was calculated considering the sum of the direct cost with mechanical, civil, and instrumentation work as well as piping and electrical wiring. The OpEx involves raw materials, utilities, maintenance, depreciation, and labor costs. The raw material cost includes the feedstock and chemical reagents costs (see Table 10). For utilities, values of 7.89 USD ton^−1^, 8.07 USD ton^−1^, 8.15 USD ton^−1^, and 0.1 USD kWh^−1^ were used for low-pressure steam, medium-pressure steam, high-pressure steam, and electricity, respectively. The cooling and process water cost was calculated using a Chemical Engineering Plant Cost Index (CEPCI) of 701.4 for 2021. The maintenance was calculated as 6% of the CapEx and the depreciation using an interest rate of 17% based on the straight-line method. Finally, the labor cost was estimated considering a wage of 5.21 USD h^−1^ for an eight-hour shift per day.

(5) $$Yield_{cellulose}=\frac{Cellulose_{WIS}}{Raw\;feedstock}\times 100\%$$

(6) $$Yield_{hemicellulose}=\frac{{\left(Oligomers_{C_{5}}+Monosaccharides_{C_{5}}\right)}_{hydrolizate}}{Raw\;feedstock}\times 100\%$$

(7) $$Yield_{Lignin}=\frac{Lignin_{hydrolyzate}}{Raw\;feedstock}\times 100\%$$

#### 3.3.2. Environmental Assessment

The environmental analysis was carried out based on the indicators established by the Environmental Protection Agency (EPA). The Waste Reduction Algorithm (WAR) software (Environmental Protection Agency, USA) calculated the potential environmental impact (PEI), considering only the pretreatment stage as the control volume. Thus, the PEI was calculated as the difference between the environmental impact generated by the process input and output streams through five impact categories: terrestrial toxicity potential (TTP), human toxicity potential by inhalation or dermal exposure (HTPE), human toxicity potential by ingestion (HTPI), smog formation or photochemical oxidation potential (PCOP), and acidification potential or acid rain (AP). 

#### 3.3.3. Social Assessment

Social analysis is considered one of the three fundamental pillars in determining the sustainability of a process. Therefore, this work proposed an analysis to determine the social impact of implementing pretreatment schemes for rice husks related to the local community. It is important to point out that other categories associated with workers, value chain agents, and consumers were excluded from the analysis since they could not be identified and evaluated. For example, a life cycle analysis that includes the agronomic and transport stages of rice husks is not carried out, hindering the analysis of employees in the value chain. Therefore, the scope of the social analysis also involved pretreatment schemes from raw material intake to filtration for obtaining the WIS and the hydrolysate. In this work, it was assumed that two operators would be working in the pretreatment stage; therefore, an analysis of employment generated was not performed since it would be constant for all the schemes. It has previously been reported that two employees can be assumed for each processing section, involving raw material and reagent reception, pretreatment, reaction, and separation [82]. Table 11 summarizes the stakeholders, subcategories and indicators used to evaluate the social impact of the different rice husk pretreatment systems. All indicators were normalized using statistics and information derived from the industrial sector in the Colombian context. Additionally, the methodology for the social analysis was carried out following the Product Social Life Cycle Assessment (PSILCA) database developed by GreenDelta [83].

##### Stakeholder: Local Community

The social impact caused by the pretreatment schemes was evaluated by considering a subcategory related to the use of natural resources, energy demand, and fossil fuel extraction. Thus, the first two indicators associated with using natural resources aim to assess the level of water use in the industrial sector and the renewable available in the country. For the water use in the industrial sector, the flow used in each scheme (cooling water and process water) and the total water used at the national scale in the industrial sector are correlated. Furthermore, for the renewable water available in the country, the flow used in each scheme is related to the total water available at the national scale. The AQUASTAT database from the Food and Agriculture Organization of the United Nations (FAO) was used to calculate these indicators [84]. On the other hand, the energy demand indicator relates the energy of each scheme with the national energy demand associated with the industrial sector. For the calculation of this indicator, the annual reports of the Mining-Energy Planning Unit (UPME) were used [85]. Finally, the fossil fuel extraction indicator is understood as the diesel energy (fuel for a steam boiler) required based on the thermal demand of low, medium, and high-pressure steams in the pretreatment schemes. This indicator also considers the UPME annual reports. The equations for calculating the social indicators are summarized in Table 12.

## 4. Conclusions

This work demonstrated the definition of pretreatment efficacy in biorefinery schemes assessed through ten sustainable indicators involving operational considerations, cost-effectiveness, environmental impact, and social issues, described as follows: (i) lignocellulosic fraction removal; (ii) accessibility index; (iii) formation of by-products or hydrolyzed products; (iv) formation of inhibitory compounds; (v) technology readiness level; (vi) energy demand; (vii) capital costs; (viii) operating costs; (ix) environmental impact; and (x) social impact. Through an in-depth literature review and process simulation, it was possible to identify the best pretreatment schemes for individual cellulose isolation and the removal of hemicellulose and lignin. It was concluded that at a preliminary analysis, the best pretreatments for cellulose isolation in the WIS are dilute acid, wet air oxidation, or organosolv. On the other hand, if biorefineries are planned to valorize the hemicellulose fraction, it is recommended to implement LHW, steam explosion, or dilute acid pretreatments. In lignin-based biorefineries, it is proposed to use kraft, alkali, or LHW pretreatments. As a main result, an approximation of sustainable pretreatments in the Colombian context is described due to the assessment of techno-energetic, economic, environmental, and social indicators.. These results would help future work on designing complex biorefineries to choose the best pretreatment scheme as it drastically influences the reaction and downstream processes for product separation.

## Figures and Tables

**Table 4:** Technical analysis of pretreatments to produce different lignocellulosic fractions.

Lignocellulosic Fraction	Pretreatment	Yield (kg 100 kg^−1^) ****	Utilities		
			Cooling Water (ton h^−1^)	Steam (ton h^−1^)	Electricity (kW)
Cellulose	Wet oxidation	20.9	4220	59.6 ***	147.6
	RAAE	20.2	5260	547.7 *	8589.0
	Organosolv	18.1	22,200	190.4 * 25.8 **	226.3
	Diluted acid	20.3	1580	21.2 ***	9.4
	Kraft	20.2	1580	33.3 **	151.8
	Ionic liquid	18.9	3720	247.3 * 7.8 **	361.9
Hemicellulose	Steam explosion	9.9	161	1.1 ***	61.20
	LHW	11.5	1500	18.4 **	111.8
	Ionic liquid	0.7	327	247.3 ** 7.8 **	361.9
	Organosolv	12.2	22,100	25.4 **	308.9
	Diluted acid	12.8	1540	21.2 ***	109.3
	ARP	8.1	-	4.1 **	105.1
Lignin	Alkali	14.2	5860	69.4 **	236.8
	Kraft	21.6	1220	16.9 **	122.8
	Ionic liquid	3.4	244	1.3 * 7.4 ***	264.3
	LHW	13.9	136	4.1 ***	64.3
	Organosolv	13.9	22,100	190.2 * 25.4 **	308.9
	RAAE	15.9	5260	547.7 *	8589.0

* Low-pressure steam; ** medium-pressure steam; *** high-pressure steam; **** yield based on the isolated lignocellulosic fraction per initial raw material (50 tons d^−1^). For cellulose, this is the six-carbon fraction of the WIS. For hemicellulose, this corresponds to the oligomers and monosaccharides of five carbons in the hydrolysate. For lignin, this is the solubilized lignin. See Equations (5)–(7).

**Table 5:** Economic assessment of pretreatments to produce different lignocellulosic fractions.

Lignocellulosic Fraction	Pretreatment	CapEx (M-USD)	OpEx (M-USD Year^−1^)				
			Raw Materials	Utilities	Depreciation	Others *	Total
Cellulose	Wet oxidation	2.46	0.63	6.76	0.58	0.16	8.13
	RAAE	6.65	1296.46	48.23	1.57	0.41	1346.67
	Organosolv	3.73	40.54	25.28	0.88	0.24	66.93
	Diluted acid	0.81	0.79	2.83	0.19	0.06	3.88
	Kraft	2.25	764.03	3.73	0.53	0.15	768.44
	Ionic liquid	1.96	6603.88	18.67	0.46	0.13	6623.14
Hemicellulose	Steam explosion	0.63	0.44	0.75	0.15	0.05	1.39
	LHW	0.88	0.41	2.60	0.21	0.06	3.28
	Ionic liquid	2.45	6603.88	18.66	0.58	0.16	6623.27
	Organosolv	4.09	40.76	15.80	0.97	0.26	67.30
	Diluted acid	0.96	0.79	2.83	0.23	0.07	3.92
	ARP	1.12	0.89	0.38	0.26	0.08	1.61
Lignin	Alkali	1.62	41.60	8.19	0.38	0.11	50.28
	Kraft	0.99	1.20	2.38	0.23	0.07	3.89
	Ionic liquid	2.15	197.47	1.51	0.51	0.14	199.63
	LHW	0.60	0.37	0.96	0.14	0.05	1.52
	Organosolv	4.09	40.54	25.29	0.97	0.26	67.05
	RAAE	6.45	1181.08	48.23	1.52	0.40	1231.23

* Maintenance and labor costs.

**Table 6:** Social indicator results of access to material resources adapted to the Colombian context.

Lignocellulosic Fraction	Pretreatment	$FWU_{sector}$ (%)	$FWU_{country}$ (%)	ED (%)	EF (%)
Cellulose	Wet oxidation	1.00	1.74 × 10^−3^	2.03 × 10^−4^	1.71 × 10^−5^
	RAAE	3.27	0.32 × 10^−3^	0.01	1.53 × 10^−4^
	Organosolv	5.20	9.05 × 10^−3^	3.11 × 10^−4^	6.06 × 10^−5^
	Diluted acid	0.37	6.51 × 10^−4^	1.29 × 10^−4^	6.09 × 10^−6^
	Kraft	5.62	0.10 × 10^−3^	2.08 × 10^−4^	9.56 × 10^−6^
	Ionic liquid	0.13	2.21 × 10^−4^	4.97 × 10^−4^	7.14 × 10^−5^
Hemicellulose	Steam explosion	0.04	6.63 × 10^−5^	2.03 × 10^−4^	3.07 × 10^−7^
	LHW	0.36	6.19 × 10^−4^	0.01	5.27 × 10^−6^
	Ionic liquids	0.13	2.18 × 10^−4^	3.11 × 10^−4^	7.14 × 10^−5^
	Organosolv	5.20	9.04 × 10^−3^	1.29 × 10^−4^	6.05 × 10^−5^
	Diluted acid	0.36	6.33 × 10^−4^	2.08 × 10^−4^	6.09 × 10^−6^
	ARP	0.21	9.47 × 10^−7^	4.97 × 10^−4^	1.17 × 10^−6^
Lignin	Alkali	1.39	2.42 × 10^−3^	2.03 × 10^−4^	1.99 × 10^−5^
	Kraft	0.29	5.00 × 10^−4^	0.01	4.85 × 10^−6^
	Ionic liquid	0.06	9.98 × 10^−5^	3.11 × 10^−4^	2.48 × 10^−6^
	LHW	0.03	5.60 × 10^−5^	1.29 × 10^−4^	1.18 × 10^−6^
	Organosolv	5.19	9.03 × 10^−3^	2.08 × 10^−4^	6.05 × 10^−5^
	RAAE	1.46	2.55 × 10^−3^	4.97 × 10^−4^	1.53 × 10^−4^

$FWU_{sector}$: level of industrial water use (withdrawal), $FWU_{country}$: level of industrial water use (renewable), ED: energy demand, EF: extraction of fossil fuels.

**Table 7:** Pretreatment efficacy by heuristic analysis.

**Lignocellulosic Fraction**	**Pretreatment**	**Evaluative Indicator**											
		$R_{I}$	$A_{I}$	**By-Products**	**Inhibitors**	${TRL}_{I}$	$E_{I}$	**CapEx**	**OpEx**	PEI	$SI_{I}$	**Total Using** $\omega_{1}$	**Total Using** $\omega_{2}$
		**Weight Factor (** $\omega_{1}$ %) *											
		10	10	10	10	10	10	10	10	10	10	100	-
		**Weight Factor (** $\omega_{2}$ %) **											
		8	10	8	10	12	11	12	12	15	3	-	100
Cellulose	Wet oxidation	9	6	5	8	10	5	4	7	6	8	6.8	6.7
	RAAE	8	8	6	7	8	1	3	2	0	8	5.1	4.5
	Organosolv	9	8	8	6	4	3	5	6	9	6	6.3	6.3
	Diluted acid	9	6	7	4	9	7	8	8	6	8	7.2	7.1
	Kraft	9	4	7	7	4	6	5	3	6	6	5.8	5.6
	Ionic liquid	9	9	4	7	8	2	4	1	7	8	5.9	5.6
Hemicellulose	LHW	7	8	8	5	8	8	7	8	8	7	7.7	7.6
	Organosolv	6	7	7	5	3	3	9	8	6	6	6.4	6.2
	Ionic liquids	8	6	7	3	5	1	7	6	6	8	5.7	5.5
	Dilute acid	7	4	8	5	8	9	7	8	7	7	7.1	7.1
	ARP	7	7	4	6	8	8	7	6	7	7	6.8	6.9
	Steam explosion	7	7	9	8	8	8	8	8	8	7	7.5	7.5
Lignin	Alkali	6	8	7	9	10	5	8	6	7	8	7.4	7.4
	Organosolv	6	4	6	6	8	2	4	5	8	8	5.7	5.6
	Kraft	10	7	7	9	10	6	8	8	7	6	7.7	7.8
	LHW	7	3	7	7	8	8	8	8	8	8	7.2	7.2
	Ionic liquid	9	5	7	8	4	8	5	3	8	6	6.4	6.3
	RAAE	9	9	6	8	4	1	2	1	7	8	5.6	5.2

* Weighting using equal distribution of 10%; ** weighting using statistical analysis.

**Table 8:** Indicator scale for the technology readiness level indicator ($TRL_{I}$).

Scale	Description	Group
1	Fundamental research	Research
2	Technology formulation	
3	Applied research (proof of concept)	
4	Small-scale development (laboratory scale)	Development
5	Scale-up development (pilot scale)	
6	Full-scale development	
7	System validated in simulation	Innovation
8	System validated in real life	
9	Commercial application	

**Table 9:** Physicochemical characterization of rice husk.

Parameter	Mass Composition (g 100 g^−1^) on a Dry Basis
Initial moisture	12.01
Cellulose	29.34
Hemicellulose	15.02
Lignin	29.14
Total extract	7.86
Fats	3.80
Protein	1.29
Pectin	13.55
Ash	18.52

**Table 10:** Feedstock and chemical reagent cost.

Input	Cost (USD ton^−1^)	Reference
Rice husk	20	Colombian regional market
Sodium carbonate	234	Means of Alibaba *
Ammonia	450	
Ethanol	863	Colombian regional market
Sulfuric acid	94	Means of Alibaba *
Sodium hydroxide	450	
Sodium sulfide	350	
Ionic liquid	13,500	

* Cost calculated as a mean of www.alibaba.com (accessed on 20 November 2022).

**Table 11:** Social indicators used to evaluate the social impact of pretreatment schemes.

Stakeholder	Subcategory	Indicator	Unit
Local community	Access to material resources	Level of industrial water use (withdrawal) Level of industrial water use (renewable) Energy demand Extraction of fossil fuels	% % % %

**Table 12:** Social indicators used to evaluate the social impact.

Indicator	Equation
Level of industrial water use (withdrawal)	$FWU_{sector}=\frac{W_{process}+W_{cooling}}{W_{withdrawal}\;by\;industry\;sector\;in\;Colombia}$
Level of industrial water use (renewable)	$FWU_{country}=\frac{W_{process}+W_{cooling}}{W_{renewable}\;in\;Colombia}$
Energy demand	$ED=\frac{Energy\;demand\;in\;process}{Energy\;demand\;in\;Colombia}$
Extraction of fossil fuels	$EF=\frac{Dieselenergy}{National\;energy\;demand\;(diesel)}$

## Data Availability

No new data were created or analyzed in this study. Data sharing is not applicable to this article.
